# Seasonal patterns of malaria, genital infection, nutritional and iron status in non-pregnant and pregnant adolescents in Burkina Faso: a secondary analysis of trial data

**DOI:** 10.1186/s12889-021-11819-0

**Published:** 2021-09-27

**Authors:** Stephen A. Roberts, Loretta Brabin, Halidou Tinto, Sabine Gies, Salou Diallo, Bernard Brabin

**Affiliations:** 1 Division of Population Health, Health Services Research and Primary Care, Faculty of Biology, Medicine and Health, University of Manchester, Manchester Academic Health Science Centre (MAHSC), Oxford Road, Manchester, M139PL UK; 2 Clinical Research Unit of Nanoro, (IRSS—URCN), B.P.218, Ouagadougou, 11 Burkina Faso; 3 Department of Biomedical Sciences, Prince Leopold Institute of Tropical Medicine, Antwerp, Belgium; 4 Medical Mission Institute, 97074 Würzburg, Germany; 5 Liverpool School of Tropical Medicine and Institute of Infection and Global Health, University of Liverpool, Liverpool, L7 3EA UK; 6 Global Child Health Group, Academic Medical Centre, University of Amsterdam, Amsterdam, The Netherlands

**Keywords:** Season, Adolescents, iron biomarkers, Malaria, Abnormal vaginal flora, Bacterial vaginosis, Body mass index, MUAC, Burkina Faso

## Abstract

**Background:**

Adolescents are considered at high risk of developing iron deficiency. Studies in children indicate that the prevalence of iron deficiency increased with malaria transmission, suggesting malaria seasonally may drive iron deficiency. This paper examines monthly seasonal infection patterns of malaria, abnormal vaginal flora, chorioamnionitis, antibiotic and antimalarial prescriptions, in relation to changes in iron biomarkers and nutritional indices in adolescents living in a rural area of Burkina Faso, in order to assess the requirement for seasonal infection control and nutrition interventions.

**Methods:**

Data collected between April 2011 and January 2014 were available for an observational seasonal analysis, comprising scheduled visits for 1949 non-pregnant adolescents (≤19 years), (315 of whom subsequently became pregnant), enrolled in a randomised trial of periconceptional iron supplementation. Data from trial arms were combined. Body Iron Stores (BIS) were calculated using an internal regression for ferritin to allow for inflammation. At recruitment 11% had low BIS (< 0 mg/kg). Continuous outcomes were fitted to a mixed-effects linear model with month, age and pregnancy status as fixed effect covariates and woman as a random effect. Dichotomous infection outcomes were fitted with analogous logistic regression models.

**Results:**

Seasonal variation in malaria parasitaemia prevalence ranged between 18 and 70% in non-pregnant adolescents (*P* < 0.001), peaking at 81% in those who became pregnant. Seasonal variation occurred in antibiotic prescription rates (0.7–1.8 prescriptions/100 weekly visits, *P* < 0.001) and chorioamnionitis prevalence (range 15–68%, *P* = 0.026). Mucosal vaginal lactoferrin concentration was lower at the end of the wet season (range 2–22 μg/ml, *P* < 0.016), when chorioamnionitis was least frequent. BIS fluctuated annually by up to 53.2% per year around the mean BIS (5.1 mg/kg^2^, range 4.1–6.8 mg/kg), with low BIS (< 0 mg/kg) of 8.7% in the dry and 9.8% in the wet seasons (*P* = 0.36). Median serum transferrin receptor increased during the wet season (*P* < 0.001). Higher hepcidin concentration in the wet season corresponded with rising malaria prevalence and use of prescriptions, but with no change in BIS. Mean Body Mass Index and Mid-Upper-Arm-Circumference values peaked mid-dry season (both *P* < 0.001).

**Conclusions:**

Our analysis supports preventive treatment of malaria among adolescents 15–19 years to decrease their disease burden, especially asymptomatic malaria. As BIS were adequate in most adolescents despite seasonal malaria, a requirement for programmatic iron supplementation was not substantiated.

**Supplementary Information:**

The online version contains supplementary material available at 10.1186/s12889-021-11819-0.

## Introduction

Seasonality defines the epidemiology of malaria and is observed for almost all infectious diseases, denoting cyclical changes in exposure and host immunity [1–3]. Studies in children showed prevalence of iron deficiency increased over the malaria season, [4] but decreased when malaria transmission was interrupted by vector control, [5] suggesting malaria was seasonally driving iron deficiency. The mechanism responsible for this interaction was hepcidin. This hormone, when stimulated by inflammation due to malaria or respiratory infection in young children, restricts enteric iron absorption and, by limiting iron availability, may lead to iron deficiency [6, 7].

Whether malaria drives seasonal iron deficiency in adolescents is an important question, given their continuing exposure to malaria and multiple other infections, alongside increased iron requirements for growth and maturation. In Burkina Faso, higher body iron stores (BIS) in adolescents predicted an increased malaria risk in the following rainy season approximately 5 months later, [8] as well as in early pregnancy in those who went on to conceive [9]. Preterm birth incidence showed a striking seasonal pattern, with a 50% increased effect size in iron supplemented women [10]. An inhibitory effect of iron on nitric oxide, interfering with macrophage-mediated action against *Plasmodium* [11]*,* as well as the parasite’s own capacity to acquire iron as a means to increase pathogenicity [12], may explain the higher malaria risk with elevated iron levels. Median levels of serum hepcidin were significantly higher in the presence of malaria parasitaemia in non-pregnant and pregnant adolescents, which could reduce iron absorption to reverse some of these effects [13]. Of interest was hepcidin up-regulation with lower genital tract infection. Concentrations of lactoferrin (Lf), which binds free iron in genital mucosa, rose in the presence of lower tract infections capable of causing chorioamnionitis, and positively correlated with serum hepcidin, serum ferritin and BIS [14]. Iron deficient adolescents were more likely to have normal vaginal flora [15]. These observations suggest that infection prevalence at a given time point is confounded by prior iron deficiency or repletion, which itself may be seasonally governed.

Seasonal studies on adolescents are lacking yet understanding seasonal infection patterns should provide a basis for timely selection of interventions to reduce adolescent morbidity and mortality, especially before a first pregnancy. To date, malaria seasonality has been interpreted mostly in relation to vector parameters and age-specific parasite prevalence [16]. Seasonal malaria-iron studies conducted in young children have relied on observations recorded at the start and end of the wet season, with events not measured during interim periods or between seasons [17]. This paper examines monthly seasonal infection patterns of malaria, abnormal vaginal flora, chorioamnionitis, antibiotic and antimalarial prescriptions, in relation to changes in iron biomarkers and nutritional indices in adolescents living in a rural area of Burkina Faso, in order to assess the requirement for seasonal infection control and nutrition interventions. Data had been collected over a 32-month period, as part of a community-based randomised controlled trial in adolescents of weekly iron supplementation [13].

## Methods

The community based randomised controlled trial of iron supplements (weekly ferrous gluconate (60 mg) with folic acid (2.8 mg) as intervention or folic acid alone as control) was approved by ethical review boards at collaborating centres and registered with clinicaltrials.gov: NCT01210040 (Additional file 1). Participants were recruited as healthy, nulliparous, non-pregnant women aged 15–24 years from thirty rural villages located within the Health Demographic Surveillance System of the Clinical Research Unit in Nanoro in rural Burkina Faso, [13] and followed weekly. Individual/guardian written consents were obtained from participants at recruitment. HIV prevalence was low (< 2%). Malaria is hyperendemic, with the main rainfall (daily chance of precipitation above 20%) from May to mid-October [18]. Food shortages occur from June to August (lean season), corresponding to the early rainy season, with food abundance post-harvest around November to December [19]. All participants were treated with albendazole and praziquantel at enrolment and provided with an insecticide impregnated bed net.

Between April 2011 and January 2014 data were collected at several study assessment points and at weekly follow-up visits. The present analysis included young menarcheal women at baseline who remained non-pregnant until the end of the trial, as well as data from those who became pregnant during the 18-month supplementation period and had re-consented at entry to the pregnancy cohort (Additional file 1). They were monitored until delivery and their babies followed up to 2 years of age [20]. As there were no significant or substantive differences in iron biomarker profiles between arms at trial end-points, [13] data from both arms were pooled for the present analysis.

### Non-pregnant cohort assessments

Baseline: Recruitment extended over 9 months. Demographic data, dietary information and medical histories, including last menstrual period and age at menarche, were collected and a clinical examination performed [13]. Height (nearest mm) and weight (nearest 100 g) for Body Mass Index (BMI) and mid-upper arm circumference (mm: MUAC) were measured in duplicate. A venous blood sample (5 ml) was collected for later iron biomarker assessments [13]. Self-taken vaginal swabs were requested for bacterial vaginosis (BV), gram stain, pH and Lf assays as previously described [14, 15]. Gram stains were scored using Nugent criteria with 7–10 indicating BV, 4–6 intermediate and 0–3 normal flora.

For trial design reasons, malaria parasitaemia and haemoglobin were not measured at baseline, although women symptomatic for malaria were treated in line with government guidelines (artesunate-amodiaquine or arthemeter-lumefantrine, or rescue treatment with quinine). Clinical malaria (axillary temperature ≥ 37.5 °C and/or history of fever within last 48 h) was confirmed by rapid diagnostic test (RDT). At the weekly visits which followed recruitment, participants symptomatic with malaria or other infections were referred to local health centres for free treatment and reasons for treatment were recorded. Missed periods were followed up with a urine pregnancy test.

End Assessment (FIN): Final assessments were made after 18 months of supplementation when procedures for nutritional and iron biomarker assessments at enrolment were repeated. Malaria parasitaemia was assessed and women with positive results were treated.

### Pregnant cohort assessments

At a first antenatal visit (ANC1) scheduled for 13–16 weeks gestation, in addition to standard antenatal care (including daily iron and folic acid for all participants and administration of intermittent preventive malaria treatment at relevant gestational dates) a blood sample (5 ml) was obtained for iron biomarkers and malaria microscopy. Self-taken vaginal swabs were requested, as for the non-pregnant cohort [15]. BMI and MUAC were measured. The pregnant cohort continued with weekly follow-up and referral for free treatment for symptomatic illness until delivery, when placentae were collected, if available, to determine placental malaria and chorioamnionitis [10].

#### Laboratory procedures

These have been previously described, [13, 14] and a technical resumé is provided in Additional file 2. Blood was assayed for plasma ferritin, serum transferrin receptor (sTfR), hepcidin and C-reactive protein (CRP). A non-pregnant internal regression slope log (ferritin) against log CRP estimate was used for ferritin correction [21]. Iron stores were calculated using the regression-adjusted ferritin estimate. Low BIS were defined as zero iron stores < 0 mg/kg. BIS (mg/kg) were calculated using the equation derived by Cook et al.: body iron (mg/kg) = − [log_10_ (1000 × sTfR/ferritin) – 2.8229]/0.1207 [22]. Hepcidin assays were performed at the Department of Laboratory Medicine, Radboud University Nijmegen Medical Center, The Netherlands [23]. Malaria films using whole blood were Giemsa stained and read by two qualified microscopists. For discrepant findings (positive/negative; > two-fold difference for parasite densities ≥400/μl; > log10 if < 400/μl), a third independent reading was made. Duplicate Lf samples were processed and analysed independently. Lf was measured by ELISA and measurements standardised for duplicate samples and sample weight [14]; the geometric mean is presented here. Gram stains were sent for Nugent scoring to the Microbiology Department at Manchester University NHS Foundation Trust, UK. Abnormal flora are defined as Nugent scores 4–10.

#### Statistical analysis

The sample available was determined by the size of the trial which was sized using formal power calculations for the scheduled malaria endpoint [13], which for pregnant women was at ANC1. Continuous outcomes, based on scheduled visits, were fitted to a mixed-effects linear model with month (1 to 12), age (centred at the median age of 17 years), and pregnancy status as fixed effect covariates and woman as a random effect. This allowed correlation between repeat measurements in the same women and increased the power of the analysis by exploiting the within-women differences across seasons. Effects were plotted adjusted for age and pregnancy and represent non-pregnant women at age 17 years. 95%CI (Confidence intervals) were computed using a profile likelihood method. Amplitude of the seasonal effect was estimated as the difference between the largest and smallest monthly coefficients. The peak and nadir were determined by the months with the largest and smallest monthly coefficients. CI for the amplitude and peak/nadir months were derived using bootstrapping. Where appropriate the outcomes were log-transformed for analysis, and coefficients and amplitudes are presented on this log scale.

Dichotomous infection outcomes were fitted with analogous logistic regression models, but as there were insufficient repeat measurements to fit a random woman effect, standard fixed-effect models are presented, with coefficients expressed as odds ratios. Seasonal amplitude is presented as the difference between the event rates in the highest and lowest months, after adjusting for age and pregnancy effects.

Prescription data were aggregated into the number of prescriptions dispensed per month and fitted using a Poisson regression model. Consideration of individual level covariates was precluded as appropriate denominators were not known. The number at risk was estimated by the number of recorded contacts (weekly home plus unscheduled health centre visits) and thus approximate to person-weeks of exposure. CI for seasonal amplitude, peak and nadir months were estimated by simulating monthly totals from a Poisson distribution using the estimated rates and numbers at risk. Seasonal amplitude is presented as the difference between event rates in the highest and lowest months.

For comparison annual means were estimated as simple means (geometric means for log-transformed outcomes) of the 12 monthly coefficients. We also present standard deviations (again log-transformed where appropriate) between observations for the continuous outcomes to provide context for the observed seasonal amplitudes.

## Results

**Fig. 1:**
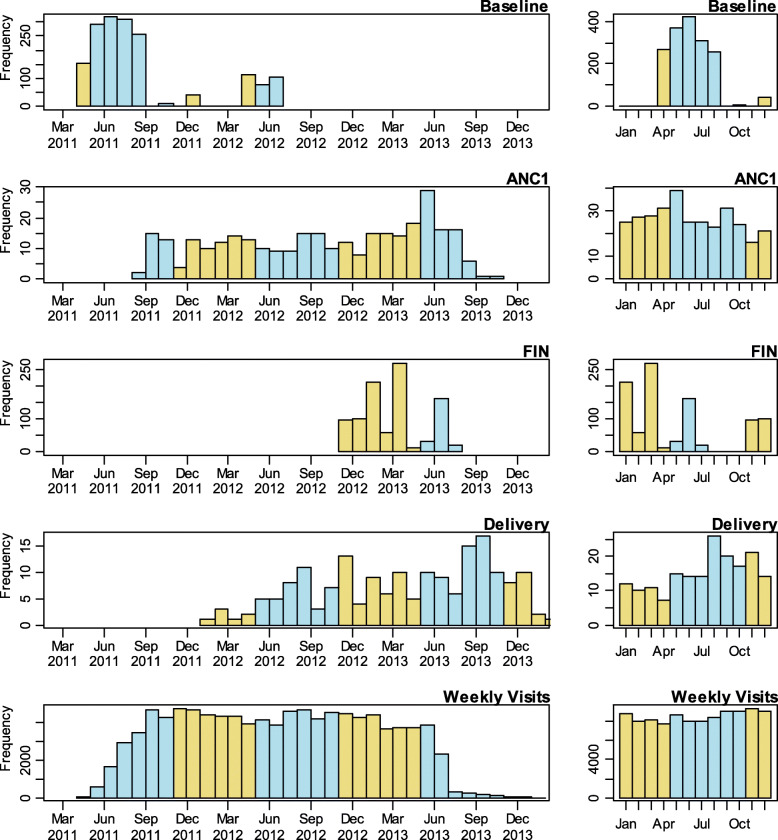
Numbers of assessments by time at the four assessment points and weekly visits. Left hand panels show the visit dates and the right hand panels the calendar months. Yellow: dry season months; blue: wet season months

Table 1 lists data sources and numbers of assessments available at baseline and end assessment for non-pregnant women, at ANC1 for pregnant women and at weekly visits. In total 1949 women contributed some data to the study. Of these 93.3% were adolescent, with only 130 more than 19 years and none less than 15 years old. All were menarcheal and nulliparous at enrolment and 315 women who became pregnant during follow-up were assessed at ANC1. At 102452 weekly follow-up visits of 1897 women, including some unscheduled visits, 1.3% were prescribed free antibiotics for mainly dysenteric symptoms, and 2.1% free antimalarials for clinical malaria. Distribution of attendances is shown in Fig. 1, with months and years of attendance for the assessment points. The total monthly attendances over 12 months were spread across wet and dry seasons. Fewer non-pregnant attendances in September and October were recorded.

**Table 1:** Data sources and sample sizes at assessment points

Outcome	Assessment points ^**a**^	Assessments	Women	Mean ^**b**^
		N	N	
Malaria parasite +ve	ANC1, FIN	1287	1230	578 (44.9)
Antimalarial Px rate/year	Weekly visits	102,452 ^c^	1897	2120 (2.1)
Antibiotic Px rate/year	Weekly visits	102,452 ^c^	1897	1287 (1.3)
Abnormal vaginal flora +ve	Baseline, ANC1, FIN	2397	1683	505 (21.1)
Chorioamnionitis +ve	Delivery	181	181	80 (44.2)
BIS, mg/kg	Baseline, ANC1, FIN	2594	1757	5.5 (2.8–7.6)
Hepcidin, nmol/l	Baseline, ANC1, FIN	2923	1885	3.8 (1.6–8.7)
sTfR, μg/ml	Baseline, ANC1, FIN	2923	1881	6.2 (5.0–7.8)
Lf, μg/ml	ANC1, FIN	1048	999	9.85 (2.85–61.17)
CRP, mg/l	Baseline, ANC1, FIN	2926	1884	0.631 (0.224–1.937)
MUAC, cms	Baseline, ANC1, FIN	2956	1898	24.1 (22.9–25.4)
BMI, kg/m^2^	Baseline, ANC1, FIN	2956	1897	20.2 (19.0–21.6)

*BIS* Body iron stores, *sTfR* Serum transferrin receptor, *Lf* Vaginal lactoferrin, *CRP* C-reactive protein, *BMI* Body Mass Index, *MUAC* Mid-upper-arm-circumference, *Px* Prescription of drug

^a^Baseline survey in non-pregnant women; end assessment survey in non-pregnant women (FIN)

First antenatal visit survey (ANC1) and weekly visits

^b^N (%) dichotomous variables; median (IQR) for continuous variables; N (rate) for prescription (Px) rate

^c^Weekly visits include some unscheduled visits

### Seasonal infection patterns

**Fig. 2:**
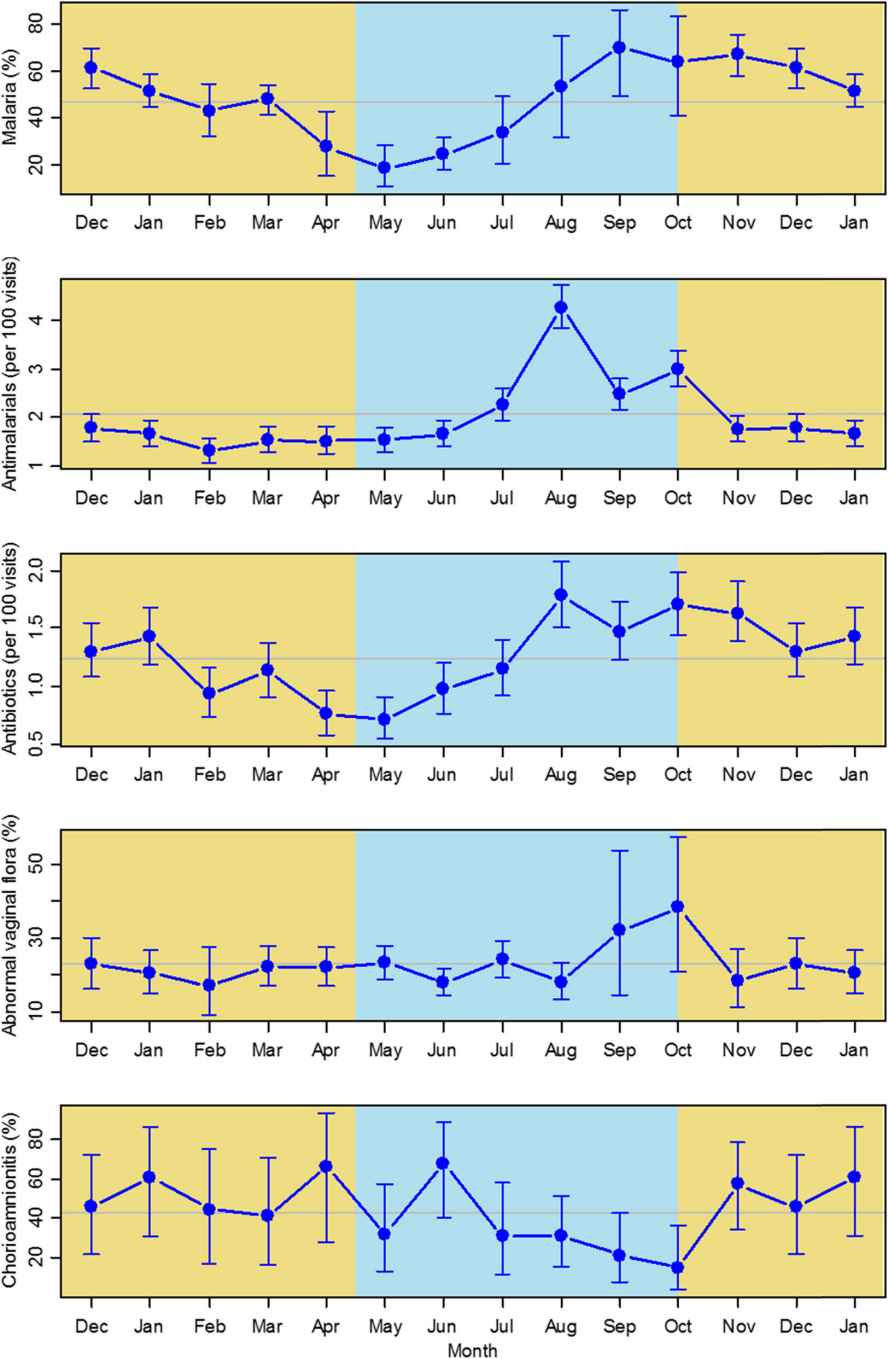
Monthly fitted estimates for infection parameters. Models fitted to all data adjusted for age and pregnancy status. Error bars represent 95% CI. Yellow: dry season months; blue: wet season months. Horizontal line is annual mean. Lines are fitted estimates and represent values for non-pregnant women of median age 17 years

Combined non-pregnant and pregnant monthly prevalence estimates for malaria parasitaemia and for genital bacterial flora with a Nugent score ≥ 4 are shown in Fig. 2, together with frequency of prescriptions for antibiotics and antimalarials. Malaria parasitaemia prevalence showed highly significant seasonal variation (*P* < 0.001), with a wet season September peak of 70% in non-pregnant women and a single dry season nadir in May of 18%. The September peak malaria prevalence for pregnant women in early pregnancy at ANC1 was 81%. The odds ratio for increased malaria infection in pregnancy was 1.9 (95%CI 1.3–2.6) (Table 2). Antimalarials for clinical cases were more frequently prescribed in the middle of the wet season with an August peak (*P* < 0.001), although malaria prevalence remained above 40% for 4 months into the dry season.

**Table 2:** Summary of seasonal models

Outcome	Mean ^**a**^	SD ^**b**^	Age		Pregnancy		Range ^**d**^	Season			
			Effect ^**c**^	***P***	Effect ^**c**^	***P***		Amplitude ^**e**^	Peak	Nadir	***P***
Malaria parasite +ve, %	0.47	–	0.85 (0.79:0.92)	< 0.001	1.87 (1.33:2.63)	< 0.001	18.3–70.1	0.52 (0.44:0.73)	Sep (Aug:Dec)	May (Apr:Jun)	< 0.001
Antimalarial Px, rate/100 weeks ^f^	2.06	–	–	–	–	–	1.3–4.3	2.96 (2.48:3.43)	Aug (Aug:Aug)	Feb (Feb:May)	< 0.001
Antibiotic Px, rate/100 weeks ^f^	1.24	–	–	–	–	–	0.7–1.8	1.07 (0.92:1.43)	Aug (Aug:Nov)	May (Apr:May)	< 0.001
Abnormal flora +ve, %	0.23	–	1.04 (0.98:1.10)	0.17	0.74 (0.52:1.03)	0.078	17.3–38.2	0.21 (0.12:0.46)	Oct (Jul:Oct)	Feb (Sep:Aug) ^g^	0.28
Chorioamnionitis +ve,%	0.43	–	1.11 (0.93:1.35)	0.26	–	–	14.8–67.6	0.53 (0.44:0.96)	Jun (Jan:Dec) ^g^	Oct (May:Mar)	0.026
BIS, mg/kg	5.09	3.82	−0.15 (−0.24:0.06)	0.001	2.06 (1.67:2.45)	< 0.001	4.10–6.81	2.71 (2.07:3.55)	Nov (Nov:Nov)	Apr (Apr:Oct)	< 0.001
Hepcidin, nmol/l ^h^	3.68	1.22	−0.08 (− 0.10:-0.05)	< 0.001	− 0.18 (− 0.32:-0.05)	0.007	2.75–5.21	0.64 (0.49:0.96)	Aug (Aug:Oct)	Jan (Oct:Apr)	< 0.001
sTtR, μg/ml ^h^	6.72	0.39	−0.00 (− 0.01:0.01)	0.61	− 0.12 (− 0.16:-0.08)	< 0.001	6.09–8.30	0.31 (0.21:0.44)	Oct (Sep:Oct)	Apr (Feb:May)	< 0.001
Lf, μg/ml ^h^	7.87	2.27	0.07 (−0.01:0.15)	0.10	1.12 (0.76:1.47)	< 0.001	2.0–21.5	2.38 (1.53:4.11)	Oct (May:Jan)	Aug (Apr:Sep)	0.016
CRP, mg/l ^h^	0.63	1.75	−0.06 (−0.10:-0.03)	< 0.001	1.82 (1.62:2.02)	< 0.001	0.41–1.28	1.14 (0.76:1.80)	Sep (Sep:Jan)	Apr (Dec:Jun)	< 0.001
BMI, kg/m^2^	19.99	2.02	0.40 (0.35:0.44)	< 0.001	0.62 (0.48:0.77)	< 0.001	19.5–20.4	0.88 (0.70:1.38)	Mar (Feb:Mar)	Oct (Sep:Dec)	< 0.001
MUAC, cm	23.98	1.94	0.37 (0.33:0.42)	< 0.001	−0.57(−0.72:-0.42)	< 0.001	23.7–24.3	0.65 (0.56:1.15)	Mar (Feb:Sep)	Nov (Sep:Dec)	< 0.001

*BIS* Body iron stores, *sTfR* Serum transferrin receptor, *Lf* Vaginal lactoferrin, *CRP* C-reactive protein, *BMI* Body Mass Index, *MUAC* mid-upper-arm-circumference, *Px* Prescription of drug

^a^Mean is value averaged over the whole year

^b^SD is the standard deviation of the raw data for the continuous variables on a log-scale for transformed outcomes to allow comparison with effect sizes and amplitude of seasonal effects

^c^Effect sizes are differences or differences in log-transformed values for continuous variables and odds ratios for dichotomous

variables. Brackets are 95% CI

^d^Minimum to maximum seasonal range. For log-transformed variables this corresponds to a proportional variation around a mean value

^e^Amplitude is the peak-nadir difference after adjusting for age and pregnancy effects. For log transformed variables this is the difference on a log-scale

^f^Antibiotics and antimalarials are unadjusted estimates (see methods)

^g^Peak/nadir not well defined due to multiple or very broad peaks/troughs

^h^Log-transformed

Prevalence of abnormal vaginal bacterial flora was uniform through the dry season with a mean value of 23%, with higher prevalence of 38% at the end of the wet season (*P* = 0.28). There were no significant effects of age or pregnancy status on prevalence of abnormal vaginal flora. Prescription of antibiotics almost doubled during the wet season (*P* < 0.001). Monthly prevalence of chorioamnionitis is shown for 181 women with delivery placental samples. Mean chorioamnionitis prevalence was 43%, with higher values through most of the dry season (peak June) and a nadir at the end of the wet season (October) (*P* = 0.026). Table 2 summarises significance levels for seasonal variation, along with pregnancy and age effect sizes.

### Seasonal iron biomarker patterns

**Fig. 3:**
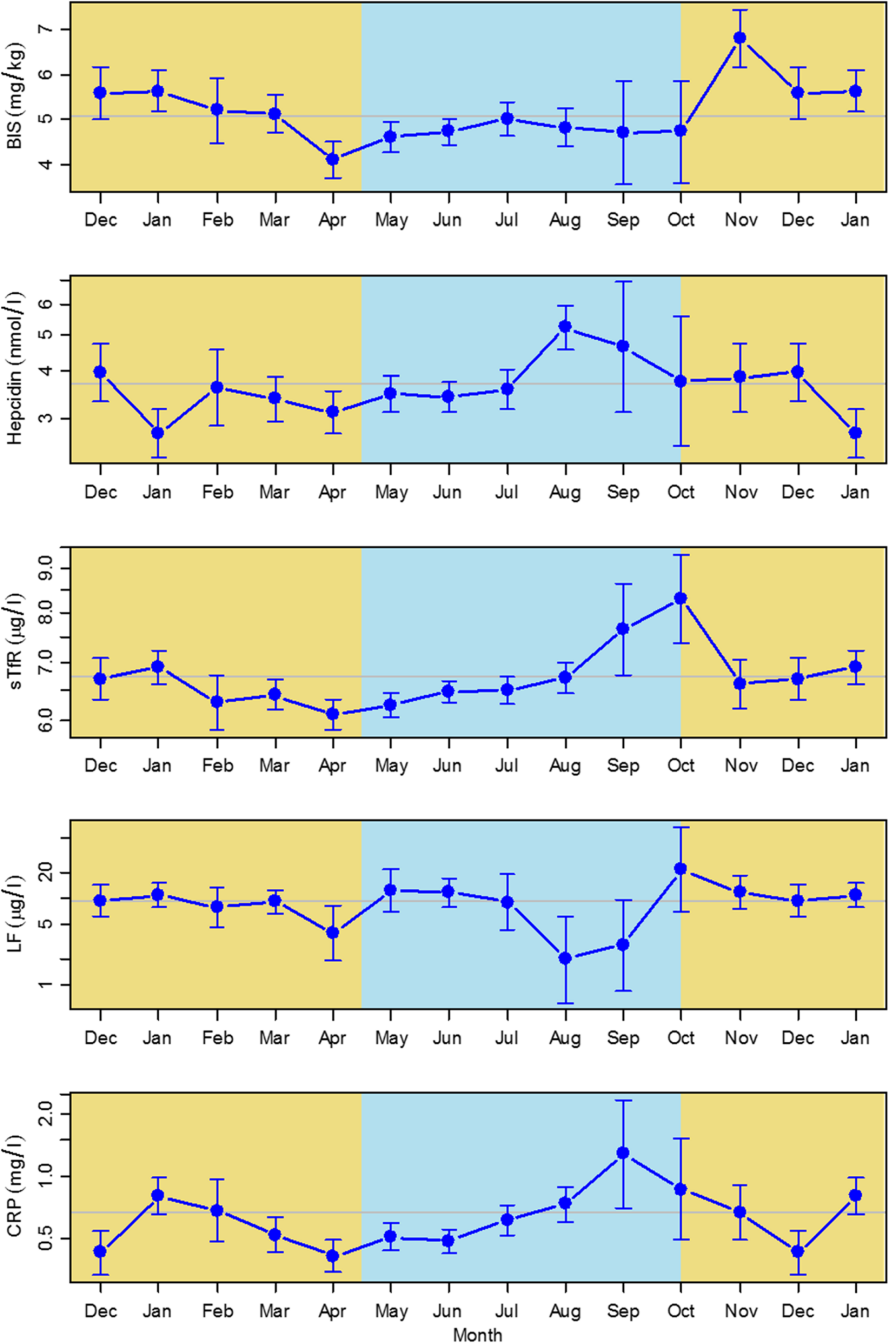
Monthly fitted estimates for iron biomarkers and C-reactive protein. Models fitted to all data adjusted for age and pregnancy status. Error bars represent 95% CI. Yellow: dry season months; blue: wet season months. Horizontal line is annual mean. Lines are fitted estimates and represent values for non-pregnant women of median age 17 years

Monthly concentrations of iron biomarkers and CRP are shown in Fig. 3, with a summary of significance levels and timing of seasonal peaks and troughs in Table 2. Median BIS were 5.1 mg/kg^2^ and showed significant seasonal variation (range 4.1–6.8 mg/kg) with a single November peak and April nadir, (*P* < 0.001). This represents an average of 53.2% variation around mean BIS during a 12-month period. BIS values were slightly below the annual mean value throughout the wet season but recovered quickly in the dry season and remained slightly above this annual mean. However, percentages with low BIS (< 0 mg/kg) were 9.5% in the dry season and 9.2% in the wet season (*P* = 0.19), with the corresponding adjusted estimates in non-pregnant women being 8.7% (dry) and 9.8% (wet) (*P* = 0.36), after fitting a logistic model allowing for age and pregnancy.

Mean sTfR concentration was 6.7 μg/ml, with a single October peak following an increase in concentration in the last 2 months of the wet season (*P* < 0.001). sTfR concentrations declined in November and December in the early dry season when malaria prevalence was still high. Monthly hepcidin concentrations were higher towards the end of the wet season compared to dry season values (*P* < 0.001). The mean value was 3.68 nmol/l, with relatively narrow range (2.75–5.21 nmol/l). A single peak in concentration occurred in August, with a single nadir in January in the middle of the dry season.

Monthly vaginal Lf concentration was almost uniform throughout the year, but with a decline in values in the late wet season and seasonal range of 2.0–21.5 μg/ml, *P* = 0.016). Seasonal effects on iron biomarkers were small compared to the variation between individuals as shown by their standard deviations (Table 2). CRP concentrations were moderately raised and showed smooth seasonal cyclic variation with a range of 0.41–1.28 mg/l, with two nadirs in the dry season and a single peak at the end of the wet season (*P* < 0.001). CI for all biomarkers were wider for September and October due to the smaller number of assessments available for these months.

### Anthropometric and dietary patterns

**Fig. 4:**
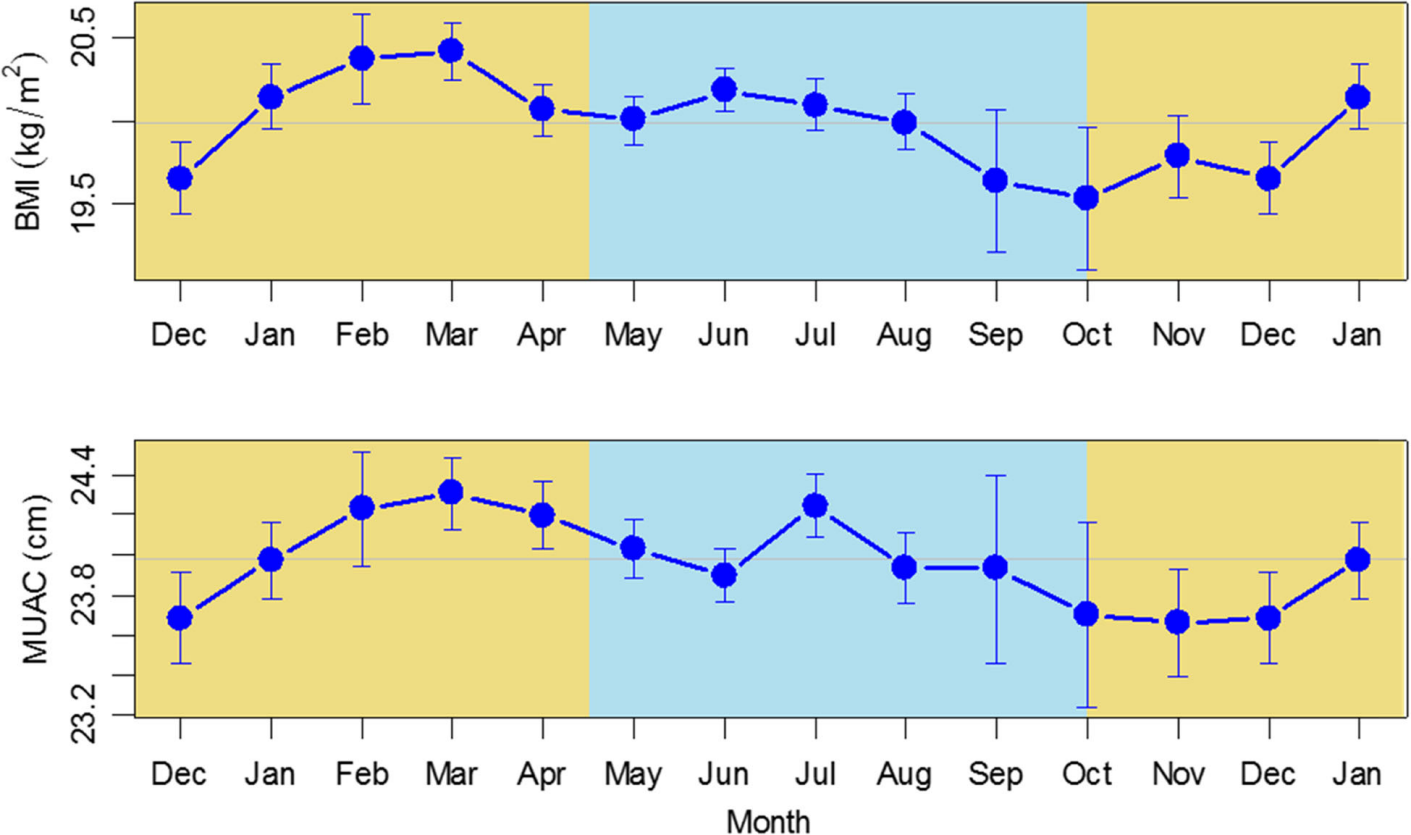
Monthly fitted estimates for BMI and MUAC. Models fitted to all data adjusted for age and pregnancy status. Error bars represent 95% CI. Yellow: dry season months; blue: wet season months. Horizontal line is annual mean. Lines are fitted estimates and represent values for non-pregnant women of median age 17 years

Monthly variation in BMI and MUAC are shown in Fig. 4, with summary statistics in Table 2. Highly significant seasonal variation was observed for both indices (*P* < 0.001). Seasonal patterns for each indicator were closely mirrored, with the same peak month (March) at the end of the post-harvest period, and nadirs in October for BMI and November for MUAC at the end of the wet season. The ranges of these changes were 19.5–20.4 kg/m^2^ for BMI and 23.7–24.3 cm for MUAC (both *P* < 0.001).

## Discussion

This study assessed seasonal trends in adolescent infections, nutritional status and risk of iron deficiency over a 32-month period. The data were consistent with malaria and other infections driving the hepcidin response to control iron homeostasis. As a result, BIS fluctuated by up to 53% per year around a mean of 5.5 mg/kg. Iron stores were below the mean in the wet season but recovered sharply post-harvest, at the beginning of the dry season when food was more plentiful and workloads decreased. Increased prescription of antibiotics and antimalarials in the wet season reflected higher infection risk, which may partly relate to reduced immunity with poorer diet, [24] and increased malaria exposure.

The finding that ~ 75% of all those followed were parasitaemic at the peak of the wet season is a clear indicator of the need to lower wet season malaria transmission in this older age group. We have previously reported a high risk of adverse birth outcomes in the same adolescents, [10] and an increased risk of malaria in their infants [20]. Elevated hepcidin values in the dry season suggest that chronic asymptomatic infections are carried over from the wet season and contribute to these outcomes. In this study, 96% of malaria infections in non-pregnant and pregnant women were asymptomatic [13]. Asymptomatic *P.falciparum* parasitaemias in young pregnant women are also described in Mali, Ghana and Gambia [25], but pregnant women are easier to reach for treatment than non-pregnant adolescents. School-based intervention programmes for 5 to 15 year old children [26–28] may be feasible, but would not cover the older age group in our study. In Burkina Faso, secondary school enrolment for both sexes in 2019 was just 43% [29]. Making services more accessible to adolescents would be helpful, especially for symptomatic gastrointestinal and respiratory problems, but asymptomatic malaria and genital infections would still be missed.

Seasonal malaria chemoprevention of children less than 6 years is recommended by the World Health Organization and is being widely implemented in community settings in West Africa, including Burkina Faso [30]. It requires monthly administration of a full course of antimalarials during the malaria season and is effective in reducing morbidity and mortality by maintaining therapeutic drug concentrations in the blood [31]. Research is needed to address the feasibility and cost effectiveness of a similar community-based approach for seasonal prevention of malaria in older adolescents, some of whom could be unknowingly pregnant. Clinicals trials would be required, keeping in mind that the antimalarial treatment recommended by the World Health Organization for first trimester pregnancy would be difficult to implement in the adolescent population, most of whom would not be pregnant. Current WHO guidelines recommend 7 days of quinine plus clindamycin, twice daily, for uncomplicated *P. falciparum* malaria during the first trimester [30]. Yet an Evidence Review Group convened by WHO’s Global Malaria Programme concluded that artemisinin combination treatments (ACTs) should be used to treat uncomplicated falciparum malaria in the first trimester [32]. Their use in any trimester in human pregnancy has not been associated with increased risk of miscarriage, stillbirth or congenital abnormality compared to those treated with quinine, or to the background population level of these outcomes [33]. Clinical trials of ACTs in non-pregnant adolescents should screen for pregnancy to indicate the level of acceptability of routine pregnancy testing and to exclude first trimester exposures with a seasonal malaria programme using ACTs.

Hepcidin levels were highest in August, a month before peak malaria prevalence, and did not rise further despite continued malaria transmission in the dry season. An inflammatory signal denoted by rising CRP concentrations at the start of the wet season was positively associated with rising hepcidin and decreasing CRP concentrations in the late wet season with lower hepcidin values. These stabilised around a mean of 3.7 nM/l (Table 2), considerably higher than the 2.6 nM/l median reference values for healthy western women (18–24 years), using the same assay [23]. In young non-pregnant Beninese women with asymptomatic parasitaemias, a ~ 40% reduction in iron absorption occurred at similar hepcidin levels to those reported here [34], and being persistently raised, would anticipate an increased risk of iron deficiency. Yet a decline in BIS levels did not occur until four to 5 months after the hepcidin peak and were lowest in April, mid-dry season. Mean BIS estimates were comparable to those reported for first trimester American women in the NHANES survey (6.28 mg/kg), and for Vietnamese non-pregnant women (4.7 mg/kg) [9].

The sTfR response was highest at the end of the wet season, a month following the peak values for malaria prevalence, CRP and hepcidin. A rise in sTfR is the initial intracellular response to a reduction in iron supply, indicating a degree of functional iron depletion and an erythropoietic response [35]. These sTfR concentrations declined in the early dry season, when malaria prevalence was still high, suggesting only a short period of malaria-related functional iron depletion, with no obvious need for ongoing iron supplementation. The problem is that iron interventions for adolescents are largely based on anaemia prevalence [36]. In a previous paper we reported an end assessment anaemia prevalence (Hb < 12 g/dl) of 43.4% in the non-pregnant cohort [9]. This is a level at which iron-containing supplementation of adolescents is recommended, including iron fortified food, multiple micronutrients, and lipid-based nutrient supplements [36]. None of these would be recommended in Burkina Faso, given the lack of an effective adolescent malaria control strategy, as well as availability of alternative dietary iron sources. Across this region, sorghum is a major contributor to dietary iron intakes across wet and dry seasons, with post-harvest crops increasing dietary diversity and providing more sources of iron [37, 38]. Traditional sorghum beer, consumed widely in Burkina Faso [39], and other areas with limited resources, also contributes to micronutrient adequacy in the diet [40].

The higher prevalence of abnormal vaginal flora in September and October was not statistically significant, although its occurrence corresponded with a significant peak in vaginal Lf concentration in October. This may indicate a local mucosal immune response to genital infection, [41] but it was not associated with hepcidin upregulation in the same months. Lf concentrations declined over the wet season as did BMI and MUAC. We have previously reported a significant positive association of vaginal Lf with MUAC and BMI in this population [14] and these nutritional indices declined throughout the wet season. Targeted seasonal measures to redress this might be warranted but relevant interventions have not been addressed [42]. General measures to improve community food security, such as reducing post-harvest food storage losses, would benefit adolescents [43]. Malaria chemoprevention should have synergistic effects in decreasing anaemia [26].

It is of interest that peak prevalence of chorioamnionitis occurred approximately 5 months following the higher prevalence of abnormal vaginal flora. Chorioamnionitis risk was highest when women were less likely to receive anti-malarials or antibiotics. Risk of chorioamnionitis may relate to early gestational exposure to abnormal flora and ascending chronic infection established in the wet season.

The main strength of this study was the large sample and weekly follow-up. Within the demographic surveillance area, all health care contacts could be readily monitored although diagnosis of dysentery was based on symptoms and not confirmed by bacteriology. The trial was not designed to assess seasonal effects, but close follow-up of participants allowed collection of infection treatment data, across clearly defined seasons, not solely at study endpoints. Haemoglobin was not measured at baseline when iron biomarkers were assessed, and dietary data were not collected at study endpoints. The rural location represented a high malaria transmission area with well-defined seasonality, common to the sub-Sahelian region. Mid-wet season data came mainly from pregnant women, as non-pregnant adolescents were generally absent from home for planting/harvesting. As a result, separate seasonal effects in pregnant and non-pregnant women could not be ascertained and constant pregnancy effects throughout the year were assumed, with seasonal effects adjusted for age and pregnancy co-variates. Geographical variations in exposures to pathogens or diet may have confounded seasonal effects as trial recruitment was conducted sequentially across villages, but these biases are likely to be negligible.

## Conclusions

Seasonal analysis is very useful for rationalising delivery of adolescent interventions. Malaria seasonal chemoprevention for this age-group should be evaluated in clinical trials to target their high prevalence of asymptomatic and chronic *P.falciparum* infections. In terms of the need for adolescent iron supplementation, this seasonal analysis showed iron stores were satisfactory at the population level and were largely maintained by host mechanisms, despite seasonal dietary changes. Some individuals would benefit from additional iron for specific clinical reasons, but this would not justify programmatic roll-out of iron supplementation. Whether improved nutrition would increase immunity to lower genital tract infections is an area for future research.

## Supplementary Information

**Additional file 1.** Background to the PALUFER safety trial of periconceptional iron supplementation.

**Additional file 2.** Resumé of laboratory methods and placental histology for chorioamnionitis.

## Data Availability

The datasets used and/or analysed during the current study are available from the corresponding author on reasonable request.

## Abbreviations

BIS: Body iron stores
sTfr: Serum transferrin receptor
BMI: Body mass index
MUAC: Mid-upper-arm-circumference
Lf: Lactoferrin
HIV: Human immunodeficiency virus
BV: Bacterial vaginosis
Px: Prescription of medication
FIN: End assessment
ANC1: First antenatal care visit
CRP: C-reactive protein
ELISA: Enzyme-linked immunosorbent assay
CI: Confidence intervals
SD: Standard deviation
IQR: Inter-quartile range
NAHNES: The National Health and Nutrition Examination Survey
PALUFER: Malaria-iron randomised iron supplementation trial
