# Supplementary material for: Seasonal patterns of malaria, genital infection, nutritional and iron status in non-pregnant and pregnant adolescents in Burkina Faso: a secondary analysis of trial data
Source: BMC Public Health. 2021 Sep 27;21:1764. doi: 10.1186/s12889-021-11819-0 (PMC8477466; doi:10.1186/s12889-021-11819-0)
Supplement: Supplementary file 1 — Additional file 1. Background to the PALUFER safety trial of periconceptional iron supplementation. [file 12889_2021_11819_MOESM1_ESM.docx]

**Additional File 1**

**Background to the PALUFER Safety Trial of Periconceptional Iron Supplementation**

**Ethics statement**

The study received ethical approvals in Burkina Faso; National Ethics Committee (CERS Ref 015-2020/CE-CM) and the Comité d’Ethique pour la Recherche en Santé du Centre Muraz; the United Kingdom Research Ethics Committee, Liverpool School of Tropical Medicine (LSTM/REC protocol 10-55); the Institutional Review Board of the Institute of Tropical Medicine (IRB/AB/AC/016) and the Antwerp University Hospital Ethics Committee, Belgium (EC/UZA). The trial was registered with Clinicaltrials.gov on 27 September 2010: Trial registration number NCT01210040. Written, informed consents were given by all individuals, with additional guardian consents provided for minors. The research was carried out in accordance with the Code of Ethics of the World Medical Association. (Declaration of Helsinki). Women were not recruited if they had clinical signs of severe anaemia (conjunctival or mucosal pallor, tachycardia, respiratory distress), or a history or presence of major clinical disease. The main results of the study were communicated to the communities at the end of the study.

**Background to the trial**

Between April 2011 and January 2014, a randomized, double blind, controlled trial was conducted amongst nulliparous, non-pregnant residents aged 15–24 years (93% adolescent, < 20 years) in a rural area of Burkina Faso experiencing hyperendemic seasonal malaria [1]. The trial design [2] and main findings have been previously reported [3-7]. Gies et al. (2018) reported that all participants received a long-lasting insecticidal net and single doses of albendazole (400 mg) and praziquantel at enrolment [3]. One cohort consisted of participants who remained non-pregnant for the duration of the trial. The second consisted of those who became pregnant during, or shortly after, the 18 month iron supplementation period.

At baseline participants were individually randomized to receive either a weekly capsule containing ferrous gluconate (60 mg) and folic acid (2.8 mg)(n=980), or an identical capsule containing folic acid alone (2.8 mg)(n=979)[3]. Field workers visited homes weekly to directly observe ingestion of capsules and monitor for pregnancy and symptoms of illness. Supplement adherence for each woman was computed. In cases of fever (temperature ≥37.5°C), or history of fever in the previous 48 hours, the field worker performed a malaria Rapid Diagnostic Test (Bioline SD, Malaria Antigen Pf 05FK50), and if positive, collected a blood sample for a thick film. RDT positives and women with symptoms of infection were referred to the Health Centre and treated at no cost with artesunate-amodiaquine for malaria and antibiotics as required for other infections, following national guidelines. As described by Brabin et al (2017), symptoms of *Trichomonas vaginalis* and bacterial vaginosis were treated with single dose metronidazole (2 g orally), and *C. albicans* with miconazole (200 mg intravaginally, daily for 3 days) [4].

**Brief summary of trial results**

The primary study end-point was malaria parasitaemia prevalence at first antenatal visit (ANC1). Secondary end-points were prevalence of anaemia, iron deficiency and lower genital tract infections at ANC1, incidence of low birthweight, preterm birth, placental malaria and chorioamnionitis. Despite adherence of 80% weekly iron did not significantly reduce iron deficiency, or anaemia prevalence at ANC1 (mean gestational age 18.5 weeks) [3], which would be consistent with chronic poor iron absorption for individuals living under high malaria pressure. Elevated serum hepcidin was more frequent in parasitaemic (22%) compared to non-parasitaemic women (11%), (P= 0.015) [3]. *Plasmodium* parasitaemia prevalence was 54.3%, at ANC1, and this prevalence did not differ by trial arm [3]. Free treatment was available for women with fever or other malaria symptoms, but most trial participants were asymptomatic (6.7% with malaria parasitaemia and fever at ANC1).

Haemoglobin was not measured at baseline as part of the trial design. In an analysis of combined study arms by Diallo et al, (2020), anaemia prevalence at end assessment in non-pregnant women was 43.4% (Hb < 12g/dl) with iron deficiency (low body iron stores <0 mg/kg) of 11.7%. In pregnant women at ANC1 69.7% (Hb < 11g/dl) were anaemic with prevalence of low body iron stores 8.0% [5].

Assessment of delivery outcomes was reported by Brabin et al, (2019) and provided evidence that long-term iron supplementation lead to excess preterm birth [6]. This was predominantly associated with the malaria transmission season, with the overall risk of delivery under 37 weeks of 27.5% compared to 13.9% in non-iron-supplemented primigravidae (adjusted P value < 0.001), with a mean gestational age five days shorter (adjusted risk difference, P = 0.012) [6]. In the control arm preterm birth incidence was consistent with estimates for other known causes such as young age, primiparity and chorioamnionitis in women not receiving periconceptional iron. Prevalence of placental malaria parasites at delivery was 33%.

Adolescents who remained non-pregnant had a parasitaemia prevalence of 41% at the end assessment, following 18 months weekly iron supplementation. Iron-supplemented non-pregnant adolescents received more antibiotic treatments for non-genital infections (P = 0.014; mainly gastrointestinal infections (P = 0.005), anti-fungal treatments for genital infections (P = 0.014) and analgesics (P = 0.008), than controls [4].

As the trial assessed the safety of iron supplementation, children born to trial mothers were followed up by Gies et al, (2020) who conducted a cross-sectional survey of these children at a mean age of nine months [7]. This showed that those born to mothers with placental malaria were at significantly higher risk of malaria infection, as were those with better iron status.

**References**

1. Rouamba T, Nakanabo-Diallo S, Derra K, Rouamba E, Kazienga A, Inoue Y, et al. Socioeconomic and environmental factors associated with malaria hotspots in the Nanoro demographic surveillance area, Burkina Faso. BMC Public Health 2019;19:249.doi:10.1186/s12889-019-6565-z. <https://www.ncbi.nlm.nih.gov/pmc/articles/PMC6396465/>
2. Brabin BJ, Gies S, Owens S, Claeys Y, D'Alessandro U, Tinto H, et al. Perspectives on the design and methodology of periconceptional nutrient supplementation trials. Trials. 2016; 17(1):58. doi: 10.1186/s13063-015-1124-0.

<https://pubmed.ncbi.nlm.nih.gov/26833080/>

1. Gies S, Diallo S, Roberts SA, Kazienga A, Powney M, Brabin L, et al. Effects of weekly iron and folic acid supplements on malaria risk in nulliparous women in Burkina Faso: A Periconceptional Double-blind Randomized Controlled Non-inferiority Trial. J Infect Dis. 2018;218(7):1099-109. doi: 10.1093/infdis/jiy257.

<https://pubmed.ncbi.nlm.nih.gov/29733403/>

1. Brabin L, Roberts SA, Gies S, Nelson A, Diallo S, Stewart CJ, et al. Effects of long-term weekly iron and folic acid supplementation on lower genital tract infection – a double blind, randomised controlled trial in Burkina Faso. BMC Medicine. 2017; 15:206 DOI 10.1186/s12916-017-0967.

https://pubmed.ncbi.nlm.nih.gov/29166928/

1. Diallo S, Roberts SA, Gies S, Rouamba T, Swinkels DW, Geurts-Moespot AJ, et al. Malaria early in the first pregnancy: potential impact of iron status. Clinical Nutrition. 2020;39(1): 204-214. doi.org/ 10.1016/j.clnu.2019.01.016.

<https://www.ncbi.nlm.nih.gov/pmc/articles/PMC6660428/pdf/nihms-1519662.pdf>

1. Brabin B, Gies S, Roberts SA, Diallo S, Lompo OM, Kazienga A, et al. Excess risk of preterm birth with periconceptional iron supplementation in a malaria endemic area: analysis of secondary data on birth outcomes in a double blind randomized controlled safety trial in Burkina Faso. Malar J. 2019:18(1):161. doi: 10.1186/s12936-019-2797-8.

<https://pubmed.ncbi.nlm.nih.gov/31060615/>

1. Gies S, Roberts SA, Diallo S, Lompo OM, Tinto H, Brabin BJ. Risk of malaria in young children after periconceptional iron supplementation. Maternal and Child Nutrition. 2020:e13106. doi:10.1111/mcn.13106.

<https://pubmed.ncbi.nlm.nih.gov/33236840/>
