# Supplementary material for: Seasonal patterns of malaria, genital infection, nutritional and iron status in non-pregnant and pregnant adolescents in Burkina Faso: a secondary analysis of trial data
Source: BMC Public Health. 2021 Sep 27;21:1764. doi: 10.1186/s12889-021-11819-0 (PMC8477466; doi:10.1186/s12889-021-11819-0)
Supplement: Supplementary file 2 — Additional file 2. Resumé of laboratory methods and placental histology for chorioamnionitis. [file 12889_2021_11819_MOESM2_ESM.docx]

**Additional File 2**

**Resumé of laboratory methods and placental histology for chorioamnionitis**

Blood samples were transported to the Clinical Research Unit at Nanoro. Clot activator tubes were centrifuged at 3000 rpm for 10 minutes and sera aliquots stored at minus 80^0^C. Ferritin and serum transferrin receptor (sTfR) were measured using duplicate sampling by ELISA (Spectro Ferritin S-22 and TFC 94 Transferrin Receptor, RAMCO Laboratories Inc, Texas). C-reactive protein assays were completed within 4-6 months of collection by ELISA (EU59131, IBL International, GMBH, Hamburg, Germany). Ranges for normal controls were 5-8 mg/ml. Intra-assay coefficients of variation were all <10%. 2. A non-pregnant internal regression slope log (ferritin) against log (CRP) estimate was used for ferritin correction, adjusting ferritin levels where CRP exceeded a reference level of the 10th centile to that reference level. Serum hepcidin was measured by competitive ELISA assay in the Department of Laboratory Medicine (TLM 830), Radboud University Medical Center, Nijmegen, The Netherlands [1]. Standards and samples were analyzed in duplicate. Samples giving readings outside the standard curve linear region were repeated at appropriate dilutions. The lower detection limit of the method was 0.26 nM. [2, 3]. The hepcidin 95% reference range for women 18-24 years from this Dutch population was: median 2.6nM; 2.5^th^ percentile 0.7nm; and 97.5^th^ percentile 10.5 nM) [2,3].

Whole blood for malaria films was stained with Giemsa and read independently by two qualified microscopists. For discrepant findings (positive/negative; > two-fold difference for parasite densities ≥400/µl; > log10 if < 400/µl), a third independent reading was made, with the mean of the two closest observations accepted.

For lactoferrin, two self-collected samples per non-menstruating woman were available. Each tube containing a single swab of vaginal eluate was weighed before and after sampling and the weight difference between initial and final weights was recorded. Samples were immediately processed and frozen at -20C°. Lf concentration was later measured by a two-site enzyme-linked immunosorbent assay (double antibody sandwich ELISA) specific to human Lf (Kamiya Biomedical Co, KT-489, Seattle, US). The intra-assay coefficient of variation was 9.7.

The derived mean sample weight was 0.033g. Residuals from a linear regression of the difference in estimated weights between the repeat samples against the difference in Lf concentration suggested a sample weight measurement error (standard deviation) of 0.035g. Mean Lf values were therefore normalised by mean weight for type of visit (allowing for pregnancy effects) derived from a linear model which included month as a covariate. A dilution series was used to assign nominal high values. Comparison of both samples helped discriminate very low Lf concentrations from samples where no vaginal fluid had been collected. [4].

**Placental sampling and storage**

Placentas were available from participants who delivered at a Health Centre or Nanoro Hospital where they could be processed after delivery to generate one section for estimating placental malaria, and a second for chorioamnionitis. For the latter, after exposing the fetal side, a biopsy of about 1cm was excised at half distance between the centre and the border of the placenta and placed into a container with formalin, as described by Brabin et al, (2019) [5]. A 10 cm x 10 cm piece of the membrane was cut with scissors and placed into the same container. Formalin fixed specimens were sent to the Department of Pathology at the National University Hospital Yalgado Ouedraogo in Ouagadougou, where tissue samples were processed by experienced technicians according to standard histopathological procedures.

Processing: Samples were embedded in paraffin wax following standard methods. Slides were prepared in duplicate to allow for different staining. De-paraffinized sections were stained with haematoxylin and eosin (H&E) and Giemsa stain.

Examination: Histological examination of all samples was done by a specialized senior pathologist with light microscopy and under polarized light. Severity of acute chorioamniotitis and funiculitis were graded histologically as early, intermediate and advanced following the Redline-classification and textbook [6,7].

**References**

1. Kroot JCC, Laarakkers CM, Geurts-Moespot A, Grebenchtchikov N, Pickkers P, van Ede AE, et al. Immunochemical and mass spectrometry-based serum hepcidin assays for a variety of iron metabolism disorders. Clin Chem 2010;56:1570-79. doi: 10.1373/clinchem.2010.149187.

<https://core.ac.uk/reader/189952120?utm_source=linkout>

1. Galesloot TE, Vermeulen SH, Geurts-Moespot AJ, Klaver SM, Kroot JJ, van Tienoven D, et al. Serum hepcidin: reference ranges and biochemical correlates in the general population. Blood 2011;117: e218-25. doi: 10.1182/blood-2011-02-337907.

[https://ashpublications.org/blood/article/117/25/e218/24315/Serum-hepcidin- reference-ranges-and-biochemical](https://ashpublications.org/blood/article/117/25/e218/24315/Serum-hepcidin-%20%20%20reference-ranges-and-biochemical)

1. Kroot, JCC, Laarakkers, CM, Geurts-Moespot, A, Grebenchtchikov, N, Pickkers, P, van Ede, AE, et al. Immunochemical and mass spectrometry-based serum hepcidin assays for a variety of iron metabolism disorders. Clin. Chem. 2010; 56:1570–1579. doi: 10.1373/clinchem.2010.149187

<https://core.ac.uk/reader/189952120?utm_source=linkout>

1. Roberts SA, Brabin L, Diallo S, Gies S, Nelson A, Stewart C, et al. Mucosal lactoferrin response to genital tract infections is associated with iron and nutritional biomarkers in young Burkinabé women. Eur J Clin Nut. 2019;doi: 10.1038/s41430-019-0444-7.

<https://www.ncbi.nlm.nih.gov/pmc/articles/PMC6842079/pdf/nihms-1529956.pdf>

1. Brabin B, Gies S, Roberts SA, Diallo S, Lompo OM, Kazienga A, et al. Excess risk of preterm birth with periconceptional iron supplementation in a malaria endemic area: analysis of secondary data on birth outcomes in a double blind randomized controlled safety trial in Burkina Faso. Malar J. 2019:18(1):161.doi:10.1186/s12936-019-2797-8. <https://pubmed.ncbi.nlm.nih.gov/31060615/>
2. Redline, RW, Clinically and biologically relevant patterns of placental inflammation. Pediatr Dev Pathol 2002;5:326-328. doi: 10.1007/s10024-002-0006-0.

<https://journals.sagepub.com/doi/10.1007/s10024-002-0006-0?url_ver=Z39.88-2003&rfr_id=ori%3Arid%3Acrossref.org&rfr_dat=cr_pub++0pubmed&>

1. Léger-Ravet M-B, Patrier S, Les infections foeto-placentaires. In Férechté E. Razavi, Dominique Carles, Pathologie Foetale et Placentaire Pratique, Chapter 15-5, Sauramps Medical, 2008.
